# Addiction Psychotherapy: Going Beyond Self-Medication

**DOI:** 10.3389/fpsyt.2022.820660

**Published:** 2022-02-09

**Authors:** Daniel Feingold, Dana Tzur Bitan

**Affiliations:** ^1^Psychology Department, Ariel University, Ariel, Israel; ^2^Shalvata Mental Health Center, Affiliated With the Sackler School of Medicine, Tel Aviv University, Tel Aviv, Israel

**Keywords:** addiction, substance use, self-medication, psychotherapy, acting out

## Abstract

Scientific and clinical work concerning the etiology of substance use and addiction has come a long way in the past decades. Current theories highlight the notion that addiction is rooted in deficits in neurobiological and psychological reward mechanisms, but also as a coping-oriented effort to contend with, or “self-medicate,” negative emotional experiences. As such, contemporary approaches in the dynamic psychotherapy of addiction highlight the compensatory nature of addiction, encouraging clinicians to detect the mental suffering underlying addiction and promote alternative coping behaviors. In this perspective article, the authors advocate for an integrative approach toward understanding and addressing addiction in psychotherapy, acknowledging its biological, psychological and social aspects. We propose that in addition to the regulatory process of self-medication, in which negative emotions are being suppressed, compulsive substance use may also reflect a substitutive function, in which negative emotions are being 'acted-out' through the use of drugs or alcohol. We suggest an integrative clinical approach which addresses these psychological aspects in a sequential manner and discuss consequent benefits for clinicians and patients working with and through addiction.

## Introduction

### Understanding Addiction: The Neuropsychological Revolution

During the past three decades, the understanding of addiction and its etiology has profoundly improved following scientific revelations concerning the neurobiological mechanisms underlying the compulsive nature of substance use, as well as new theoretical conceptualizations of the mental phenomenology and etiology of addiction (1, 2). Early social theories have associated addiction with acquired social norms of delinquency and impaired moral values. However, beginning with the 1990s, emerging evidence has pointed out that addiction is in fact associated with structural and functional brain alterations, including deficits in reward, attention, memory and motivation mechanisms (3) attributed to some extent to genetic predisposition, which is yet unspecified but appears to be shared across SUDs rather than substance-specific (2). Further research demonstrated the reciprocal nature of these neurobiological deficits, suggesting that they serve both as a cause for addiction, in predisposing individuals toward the onset of addiction, and as the effect of addiction, in further altering some neurobiological mechanisms, and even genetics, following intensive and prolonged intoxication (4). This paradigm shift led the way to a bio-ethical transformation in clinicians' view of individuals suffering from addiction. The adoption of a disease model of addiction has been manifested by its inclusion in the European and American medical classification manuals, beginning with DSM-III-R in 1987 and ICD-10 in 1992 (5).

In the field of the humanities, writers such as Eduard Khantzian and Gabor Maté have emphasized the nature of addiction as a psychologically coping-oriented mechanism, in which hurt and traumatized individuals try to cope or “self-medicate” their distress by using psychoactive substances (6, 7). According to these authors, coping-oriented substance use has a paradoxical nature: While the psychoactive effect caused by the drug may at times offer temporary relief from mental pain, in the long run suffering may be augmented by intensive substance use and its physical and psychological consequences, while natural and acquired psychological coping mechanisms are often inhibited. As recently summarized by Koob et al. in an integrative psycho-biological model for addiction, individuals enter the “addiction cycle” through deficient reward-related neural circuits and/or aversive physiological and psychological symptoms which are intensified rather than diminished by prolonged substance intake.

Notably, a substantial proportion of addiction is caused by non-substance induced negative emotions (e.g., depression, trauma), thus entrance to the 'addiction cycle' may occur via positive hedonic experience (positive reinforcement), or pursuit of relief from a withdrawal-related or emotion-related aversive experience (negative reinforcement) (8). The concept of coping-oriented self-medication process underlying the behavioral phenomenon of addiction has allowed for better treatment for individuals suffering from addiction, alongside a continuing effort to decrease the stigma associated with addiction (9). In addition to the biological and psychological roots of addiction, its social and interpersonal origins are often highlighted, with writers such as Bruce Alexander and Johan Harri stressing the social context of addiction, as a disorder of social isolation and disconnection (10, 11).

## Beyond Self-Medication

The biopsychosocial model is now a consensual etiological model in understanding addiction and more so, an emerging clinical practice implemented in many substance abuse treatment units (2). However, psychotherapists who are not specifically trained in addiction psychotherapy, or those unacquainted with this model, may focus on the self-medicating function of their patients' addiction, while undermining other important aspects of their disorder. For those clinicians, relying predominantly on self-medication in understanding and relating to addiction in psychotherapy may serve as an intuitive and pragmatic clinical framework, yet it may also be limiting due to the following reasons: (1) It often undermines the significance of impaired reward mechanisms to formation and preservation of addiction (2) it offers a somewhat narrow scope for understanding the psychological mechanisms underlying addiction, and (3) it denies clinicians a wide repertoire of interventions which may offer a therapeutic benefit while working with clients suffering from addiction.

Despite these limitations, up to date integrative theoretical frameworks for working with clients who suffer from addiction are scarce. In this paper, we propose an initial outline for an integrative model of addiction psychotherapy, based on “theoretical integration,” a concept in which several theoretical frameworks are combined to form a treatment approach (12). The case formulation described below is a composite case, synthesizing disguised information from a myriad of patients (13), which demonstrates this integrative theoretical conceptualization and clinical orientation to addiction in psychotherapy.

*Laura, A 42-year-old female patient, single with no children, was referred to an outpatient substance abuse treatment unit by her physician after suffering from symptoms of anxiety and co-occurring misuse of tranquilizers. The patient was a classical musician with a long-standing career as a renowned performer. Her father served as her professional manager, dealing with all formal aspects of her career as well as personal matters, while she was allowed to focus solely on her art. However, she reported feeling highly agitated and anxious prior to and during her performances and reported using prescription tranquilizers in increasing amounts in order to relieve her anxiety*.

### Neurobiological Deficiency

Beginning with the 1990s, emerging evidence has pointed out that addiction is in fact associated with structural and functional brain alterations, including deficits in reward, attention, memory and motivation mechanisms (3, 4). These neurobiological deficits result in a compulsive search and use of addictive substances, which is continued despite negative consequences which follow. In recent years, continuing effort is made in the field of translational science to bridge the gap between these neurobiological evidence and clinical interventions which could be used by psychotherapists in the field of addiction (2). Due to the rewarding and compensatory effects of addictive substances, patients often present an ambivalent stance toward changing their drug-related habits. Evidence has shown that motivation-focused interventions, which strive to resolve or work-through ambivalence, may be beneficial in leading the way for behavioral change (14). In the field of neuropsychological and cognitive-behavioral therapy (CBT), valuable interventions allow patients to alter their response to drug-related cues, thus promoting reduction in drug intake (15).

*Upon Admission, Laura's Predominant Mental State Was That of Preoccupation With the Drugs and Its Rewarding Psychoactive Effects. Acknowledging the Neurobiological Aspects of Her Addiction, Laura and the Therapist Successfully Engaged in Motivation-Oriented Discourse Which Allowed Laura to Enhance Her Inner Motivation for Change, Followed by Behavioral Interventions Aimed at Identifying and Avoiding cue-Induced Triggers, Increasing the use of Adaptive Reward-Related Behaviors and Managing Withdrawal and Craving*.

### Self-Medication

As recently summarized by Koob et al. (8), aversive physiological and psychological symptoms are intensified rather than diminished by prolonged substance intake, via a physical and mental withdrawal syndrome and negative emotional feedback (termed “hyperkatifeia”). According to the Ego psychology, addiction is attributed to insufficient psychological power to cope with external environment demands and/or inborn drives. For lack of such resources, emotions are rejected as they are not tolerable to the self, using the psychoactive effect of substance use (16).

*During the initial stages of psychotherapy, Laura's misuse of her medication has been efficiently identified as a self-medication for her performance anxiety and narcissistic vulnerability, which have been amplified occasionally by professional setbacks. Following this conceptualization, she acknowledged a more subtle fear which has emerged in recent years of becoming professionally irrelevant, outdated, or overmatched. Reframing her use of addictive substances as “self-medication,” or an effort to cope with and manage her fears, allowed Laura to acknowledge the underlying psychological triggers for her substance abuse and address them using cognitive restructuring*.

### Acting Out

The term “acting out” was first coined by Sigmund Freud, who identified a subtle form of defense/coping-oriented mechanism, in which an inner conflict is manifested through behavior (e.g., substance use) in order to avoid its negative emotional context (17). According to Freud and his successors, in the process of acting out, a conflictual thought (e.g., sexual desire, etc.) is substituted with a more tolerable action (18). However, enacted mental conflicts often remain unresolved and are therefore repeated compulsively. Therefore, acting out should be subject to interpretation and working-through in therapy (17).

*As Laura's treatment progressed, she began reporting reoccurring episodes of binge drug use during the weekend. During these episodes, she misused neuro-stimulants, namely by snorting of Methylphenidate (Ritalin*^*TM*^*). Laura then disclosed a history of substance abuse, predominantly misuse of prescription medications: opiates (pain killers), neuro-stimulants and hypnotics (sleeping pills), which were carefully concealed by her father as part of his guardian role, and at times of severe dependence treated discretely in private rehab centers. Processed in therapy, these binge episodes of substance use emerged as acting out of her “passion for life,” historically restrained by her parents and now extinguished by her own self-discipline. By using drugs, Laura enabled the expression of the vital parts in her inner life, which were suppressed when sober. In treatment, using Schema-focused interventions enabled Laura to decrease her self-criticism and allow herself to get in touch with the vital parts of her self without using drugs*.

Influenced by Melanie Klein's elaboration of Freud's theory, object-relation theory has emphasized psychoactive substances' role in acting out aggressive instincts toward the self and others. According to Klein, inborn aggression which is suppressed or split form the “good self” due to social taboos which forbid explicit aggressive expression, eventually lead to an emotional imbalance. Using aaptive defense mechanisms such as “sublimation” (transforming conflictual emotions into socially acceptable behaviors) or “reaction formation” (converting unconscious emotions into their opposite emotions and/or behaviors) allows individuals to regain emotional balance. However, in cases where such defense mechanisms are unavailable due to insufficient ego resources, individuals may turn to maladaptive defense mechanisms (19). In such cases, the use of psychoactive substances may allow for enactment of the suppressed aggression, either via disinhibition and explicit aggression toward other, or via self-destruction, as in the case of addiction (20). Paradoxically, in both cases substance use does not bring relief or promote equilibrium, but rather preserves a vicious cycle, in which intoxication is the root of, and the “solution” to, all suffering (21).

*In Laura's therapy, an interpersonal pattern has also emerged in the patient's binge drug intake. These episodes tended to occur at times when she felt insecure or entrapped in her relationship with her father. At time when she felt he was “abandoning” or overshadowing her in his independent professional ventures, she would act-out her separation anxiety by intensive drug intake, immediately retracting her father to the caregiver position. Inversely, at times when she felt discontent with his over-involvement and over-protectiveness, she would use drugs as enactment of her separation-individuation needs and her aggression toward his exclusive possession of a desirable good. This pattern was repeated in her stance toward the therapist, when binge substance use was at times a way to provoke him to become more engaged and attuned, when he failed to be empathic to her needs. The therapist's careful interventions regarding Laura's substance use, as often representing aggressive or rebellious acts, brought Laura relief from her self-criticism*.

## Discussion

### Integrative Model of Addiction Psychotherapy

Carefully distinguishing between the various aspects of Laura's addiction enabled broadening the scope of mutual understanding of her addiction, while expanding the repertoire of the therapist's interventions, the client's insights and alternative behavioral and emotional responses.

We propose that this initial integrative model does not rely on merely technical eclecticism (12), as it captures and addresses the multidimensional nature of addiction. A previous integrative model of addiction psychotherapy suggested integrating various aspects of treatment in a parallel manner, i.e., using various treatment modules simultaneously according to the patient's needs (22). In contrast, our model includes a sequential integration of the various therapeutic factors, in which every aspect of the biopsychosocial etiology of addiction is addressed in the appropriate time and manner. As seen in Laura's case, initial motivational enhancement allowed for a reduction in her compulsive behavior. In turn, behavioral change uncovered the psychological vulnerability underlying her addiction, leading the way for psychodynamic oriented interventions.

We believe that forming a safe and non-judgmental therapeutic alliance allows patients to enhance their inner motivation for change. Once motivation has been consolidated, behavioral goals, such as reduction in substance intake, can be achieved, gradually attenuating the neurobiological modifications caused by genetic predisposition and repeated substance use. In turn, gradual decline in the neurobiological imbalance allows patients to reflect and address the psychological and interpersonal conflicts and suffering which were suppressed or transformed by their substance use. Our model distinguishes between self-medication and acting out as two distinct psychological features of addiction. In both cases, clients and therapists should work together to uncover and address the underlying mental content, allowing for an adaptive resolution or remedy which will replace the self-sustaining substance use.

We suggest that the sequential change mechanism may in fact be a circular one, as patients often relapse to excessive substance use and it is necessary to retreat to earlier therapeutic modalities. Furthermore, we believe that biological, psychological and social aspects of addiction can be addressed in various ways; therefore our model is an inclusive one, suggesting that additional methods could be implemented in each of its phases. For example, third-wave cognitive interventions such as Acceptance and Commitment Therapy (ACT) are showing promising results (23) and should be considered legitimate means for addressing psychosocial aspects of addiction. As clinicians, we are bound to sensitively observe the diverse nature of substance use and addiction, distinguishing its manifestation as reward deficiency from its psychological and interpersonal compensatory nature. Carefully observing the various mental functions of substance use and addiction (suppression, transformation, etc.) could broaden our understanding of the psychological mechanisms underlying addiction, its phenomenology and etiology. Hopefully, this will allow for a more effective counseling for individuals who use psychoactive substances.

## Data Availability Statement

The original contributions presented in the study are included in the article/supplementary material, further inquiries can be directed to the corresponding author.

## Author Contributions

DF and DT contributed to conception, design of the study, contributed to manuscript revision, read, and approved the submitted version. DF wrote the first draft of the manuscript. Both authors contributed to the article and approved the submitted version.

## Conflict of Interest

The authors declare that the research was conducted in the absence of any commercial or financial relationships that could be construed as a potential conflict of interest.

## Publisher's Note

All claims expressed in this article are solely those of the authors and do not necessarily represent those of their affiliated organizations, or those of the publisher, the editors and the reviewers. Any product that may be evaluated in this article, or claim that may be made by its manufacturer, is not guaranteed or endorsed by the publisher.
